# Understanding Malaria Persistence: A Mixed-Methods Study on the Effectiveness of Malaria Elimination Strategies in South-Central Vietnam

**DOI:** 10.3389/fpubh.2021.742378

**Published:** 2021-12-07

**Authors:** Thuan Thi Nguyen, Charlotte Gryseels, Duong Thanh Tran, Tom Smekens, René Gerrets, Xa Xuan Nguyen, Koen Peeters Grietens

**Affiliations:** ^1^Department of Malaria Epidemiology, National Institute of Malariology, Parasitology and Entomology (NIMPE), Hanoi, Vietnam; ^2^Unit of Socio-Ecological Health Research, Department of Public Health, Institute of Tropical Medicine, Antwerp, Belgium; ^3^Faculty of Social and Behavioural Sciences, Amsterdam Institute for Social Science Research (AISSR), University of Amsterdam, Amsterdam, Netherlands; ^4^Department of Public Health, Institute of Tropical Medicine, Antwerp, Belgium; ^5^Amsterdam Institute for Global Health and Development (AIGHD), Amsterdam, Netherlands; ^6^School of Tropical Medicine and Global Health, Nagasaki University, Nagasaki, Japan

**Keywords:** malaria, malaria persistence, elimination strategies, ethnic minority, mixed methods study, Vietnam

## Abstract

Despite the scale-up of vector control, diagnosis and treatment, and health information campaigns, malaria persists in the forested areas of South-Central Vietnam, home to ethnic minority populations. A mixed-methods study using an exploratory sequential design was conducted in 10 Ra-glai villages in Bac Ai district of Ninh Thuan province to examine which social factors limited the effectiveness of the national malaria elimination strategy in the local setting. Territorial arrangements and mobility were found to directly limit the effectiveness of indoor residual spraying and long-lasting insectidical treated nets (LLINs). Households (n=410) were resettled in the “new villages” by the government, where they received brick houses (87.1%) and sufficient LLINs (97.3%). However, 97.6% of households went back to their “old villages” to continue slash-and-burn agriculture. In the old village, 48.5% of households lived in open-structured plot huts and only 5.7% of them had sufficient LLIN coverage. Household representatives believed malaria could be cured with antimalarials (57.8%), but also perceived non-malarial medicines, rituals, and vitamin supplements to be effective against malaria. Household members (*n* = 1,957) used public health services for their most recent illness (62.9%), but also reported to buy low-cost medicines from the private sector to treat fevers and discomfort as these were perceived to be the most cost-effective treatment option for slash-and-burn farmers. The study shows the relevance of understanding social factors to improve the uptake of public health interventions and calls for contextually adapted strategies for malaria elimination in ethnic minority populations in Vietnam and similar settings.

## Introduction

Although global commitment to end malaria has increased the availability of different preventive measures and treatment, the world still saw an estimate of 229 million cases and 409,000 deaths in 83 endemic countries in 2019 (1). Malaria is caused by five species of *Plasmodium* (single-celled parasites) transmitted to humans through the bites of infected female *Anopheles* mosquitoes (the vector) (2); of these five, *Plasmodium vivax (P. vivax)* and *Plasmodium falciparum (P. falciparum)* are more common, with the latter causing more severe illness and the most malaria deaths (1–3). The Greater Mekong Sub-region (GMS) consisting of Cambodia, China (Yunnan province), Lao, Myanmar, Thailand, and Vietnam has committed to a strategy to eliminate all malaria by 2030 (4, 5), leading to the scaling up of malaria interventions and a continuous decrease in malaria infections and deaths (6–8). Despite reductions at national level in GMS countries and the implementation of indoor residual spraying (IRS) and long-lasting insecticidal nets (LLINs), residual (forest) malaria transmission persists in remote forested areas and border zones challenging regional, national, and international public health interventions (6, 9, 10).

Malaria elimination in the GMS is particularly complex because the region has ecological and climatic conditions favorable to diverse vector species (10). Rapid changes in forest land use and human resettlements in the past decades have contributed to varied spatial distribution and survival of the vector population as well as to heterogeneous vector behavior (11–13). *Anopheles dirus*, one of the two main vectors in the region, feeds indoors (endophagic) and outdoors (exophagic), and rests indoors (endophilic) and outdoors (exophilic) (10, 12). This behavior means the vector can evade vector control measures such as IRS and LLINs. In addition, the National Malaria Control Programs (NMCPs) have also faced the challenge of managing an increasing burden of vivax infections, which requires more complex diagnostic tools and treatment (14–20).

In the last two decades, the Vietnamese NMCP has followed the WHO's technical guidance on malaria control by providing vector control tools (IRS and LLINs), free diagnostics and treatment to at risk populations combined with health information-education-communication (IEC) campaigns to boost the uptake of interventions (21). Effectiveness of interventions is monitored by a national disease surveillance system. Despite the success of this national strategy to control and even interrupt malaria transmission in some of the northern and southern provinces, malaria persists in specific forested areas in the South and Central regions where infections are concentrated among impoverished groups of the population, such as ethnic minority and migrant workers (22–29). Due to the sylvatic nature of *Anopheles dirus*, human activities in the forest were significantly associated with a higher malaria risk, particularly when staying overnight in the forest (14, 30–32). In endemic areas in Vietnam and similar GMS settings, the risk groups often present different demographic characteristics, risk behavior and health seeking behavior than the majority population (33–38). A mixed-methods study using exploratory sequential design was conducted in 10 villages of Bac Ai district of Ninh Thuan province in South-Central Vietnam to examine which social factors limited the effectiveness of on-going malaria elimination strategies.

## Materials and Methods

### Study Site and Population

Bac Ai district consists of nine communes with a total population of 26,440 inhabitants, according to the 2018 census (39). More than 90% of inhabitants in the study area were of Ra-glai ethnicity, the remaining being mostly of Kinh (the dominant ethnic group in Vietnam) and Cham. Declared an ethnic minority by the Vietnamese government, the matrilineally organized Ra-glai practice slash-and-burn agriculture in the hilly forested region they inhabit. Their traditional social organization and economic practices are the focus of the Vietnamese government's development interventions, aiming to integrate the Ra-glai into mainstream Kinh culture and society (40, 41).

Malaria in Bac Ai is often referred to as “forest malaria,” because malaria risk for an individual working in the forest was estimated to be three times higher than for other Ra-glai (14). Empirical evidence confirms the presence of *Anopheles dirus* in the district. These mosquitoes bite mostly between 21:00 and 05:00, causing evening activities in the forest to be of very high risk (42). In the past two decades, free-of-charge malaria services including the distribution of IRS, LLINs, long-lasting insecticide-treated hammock nets (LLIHs), health IEC campaigns, diagnostic testing, and antimalarial treatment have been provided to the population. Local surveillance data shows a strong decline in malaria incidence in recent years, from 791 confirmed malaria cases in 2010 to 53 cases in 2019 (43). During this period, the annual number of *P. falciparum* cases was approximately three times the number of *P. vivax* cases. Most malaria cases occurred during the rainy season, with two peaks from April to June, and from September to November.

### Study Design

**Figure 1:**
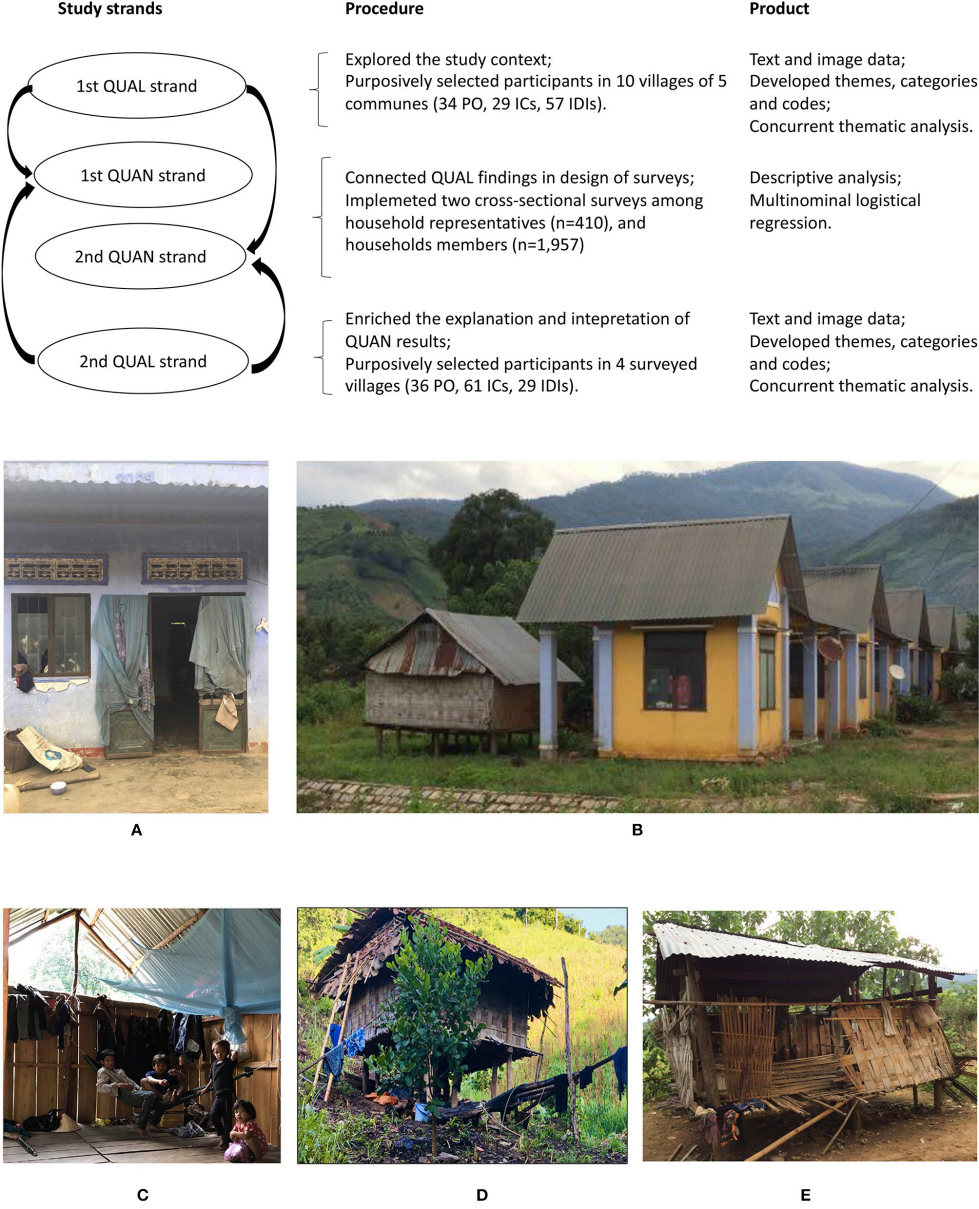
Visual model of the exploratory sequential mixed-methods study design. **(A)** An abandoned brick house in the new village, **(B)** Brick houses with additional stilt-houses, **(C)** Open-eave feature of the stilt house, **(D)** A Ra-glai traditional stilt house in the traditional territory, and **(E)** Temporary shelter in the traditional territory.

This study used an exploratory sequential mixed-methods research design (Figure 1) consisting of two qualitative and two quantitative strands for triangulation and complementarity purposes (in standard annotation: [QUAL -> QUAN -> quan -> qual]) (44). The study design prioritized qualitative research to assess the complexity of malaria persistence. The qualitative research was conducted in 10 villages (of five communes) with diverse characteristics including different malaria incidence, population size, and distance to the nearest public health facilities. The quantitative strands were conducted in four villages (of three communes), the remaining malarious villages where malaria cases were reported in five consecutive years prior to the surveys. Findings from the first qualitative strand were used to inform the design of the questionnaires used in the quantitative strands. After the analysis of the quantitative data, a second explanatory qualitative strand was conducted to further interpret and explain quantitative results.

#### Qualitative Strands

##### Rationale

The qualitative strands sought to understand the local socio-economic characteristics and how these related to the main assumptions incorporated in the local malaria elimination strategies. The researcher started with open research questions and then focused on key themes related to malaria persistence. The qualitative strands used an ethnographic approach including prolonged stays in the local setting for the researcher to get familiarized with the study population and reduce socially desirable bias.

##### Data Collection

Four periods of fieldwork (each lasting from three to six weeks) were conducted between July 2016 and May 2018. Collected data were participant observation (PO), informal conversations (ICs), group interviews (GIs), and in-depth interviews (IDIs). Interviews were conducted at people's home or at locations of their choice. Raw data were written as fieldnotes, then computerized, and stored in password-protected devices. The use of audio-recording was limited in interviews as it was frequently interpreted as a reason for mistrust by the study population. The data collection process was iterative, incorporating emerging questions and themes during fieldwork.

##### Sampling

The sampling strategy was based on a combination of existing social theory, including the PASS-model on health-seeking behavior and access to care, and emerging results (45). The selection of participants was purposive whereby the researcher spent time to build trust and rapport with people and gradually included them in the study sample. Participants with different socio-economic status, professions, ages, living locations, religions, and ethnicities were included in the sample to achieve maximum variation.

##### Analysis

Preliminary data analysis was conducted during fieldwork whereby new themes and theoretical ideas were constantly added and tested until saturation. A retroductive approach to data analysis was used with raw data being assigned to meaning units for further coding and categorization in an emergent analytical framework. Qualitative data analysis was using NVivo 12 (QSR International Pty Ltd., Cardigan, UK).

#### Quantitative Strands

##### Rationale

The quantitative strands consisted of two cross-sectional surveys. The first survey was administered to household representatives (HHRs) and focused on territory, exposure to malaria vectors including housing and LLIN ownership, and knowledge of malaria. The second survey was administered to household members (HHMs). It aimed to quantify participant's mobility, territoriality, individual risk factors, exposure to the vector in the early morning (before 07:00) and evening h (after 21:00), and their uptake of malaria services. Territoriality refers to territorial human behavior in relation to land ownership, access to resources, social and geographical space (46). In this study, territoriality was operationalized and measured through the components of mobility, living spaces, ownership of the forest field, and the variance in LLIN use and uptake of public health services.

##### Data Collection

Data were collected via surveys from November to December 2018. Two structured paper-based questionnaires were developed, field-tested, and finalized in both Vietnamese and English languages. Interviews were conducted in Vietnamese language with the help of a Ra-glai translator when needed.

##### Sampling

The surveys aimed to include everyone living in selected villages. The local census conducted in 2017 was used as the sampling frame. One week before the surveys, an invitation to participate was communicated to households through village meetings and using the public speaker system. During the surveys, up to three reminders were communicated to households by the village health workers to ensure people were informed about their chance to participate. HHRs were 16 year-old or older participants who were nominated by their household to answer the first survey questionnaire. HHMs were participants from the rest of participating households. Blood samples were collected from all participants to confirm their malaria status. Malaria tests were confirmed using light microscopy and rapid diagnostic test (RDT SD Bioline Malaria Ag P.f/Pan, Abbott, Chicago, IL, USA).

##### Analysis

Completed questionnaires were computerized and cleaned using Epi Info 7 (Centers for Disease Control and Prevention, Atlanta, GA, USA). Stata 12 (StataCorp LP, College Station, TX, USA) was used for descriptive analysis while further analysis was implemented using R (The R Foundation for Statistical Computing, Vienna, Austria). The outcome variable was reported bednet use the week before the survey comparing participants who “usually” used the bednet to those who “sometimes” or “never” used them. Bednet use the night before the survey was not selected as the outcome variable due to expected socially desirable responses. Multinomial logistic regression was used to model the outcome variable taking “usually” as the reference category which therefore expresses explanatory variables as risk factors for inconsistent (either “sometimes” or “never”) bednet use. Seven explanatory factors were identified including age (as a linear predictor, expressed on the scale of decades), sex, education, frequency of sleeping in the new village, sleeping in a stilt house as opposed to a brick house, slash-and-burn agricultural practices, and deep forest activities. Both crude (one predictor at the time) and adjusted odds ratios were reported for two arms of the multinomial logistic model. The hierarchical nature of the data, whereby HHMs were grouped into households and households were grouped into communes, was taken into account by modeling random effects with two levels of grouping. The village level was excluded because the model did not completely converge in the presence of all three levels. We chose a Bayesian approach to more reliably estimate the random effect variances and to easily gauge the amount of uncertainty around them. The priors used were those configured as the default in the R package brms (47): for the fixed effects, non-informative flat priors, and for the standard deviations of the random effects, weakly informative priors in the form of Student's T distributions with 3 degrees of freedom and a scale value of 2.5.

#### Ethics

The study protocol was jointly reviewed and approved by Ethical Review Boards of the Institute of Tropical Medicine in Antwerp (IRB/AB/ac/116), the National Institute of Malariology, Parasitology and Entomology in Hanoi (NIMPE), and the Vietnamese Ministry of Health (Decision 3731, QD-BYT). Before data collection, local authorities, representatives of the study villages, traditional leaders, and community health workers (CHWs) were sensitized about the study protocol. Participants in the quantitative strands who were 16 year-old and older were asked to consent to participate before answering the survey questionnaires. Parents or guardians of minors (those who were under 16 year-old) were asked to give consent on behalf of the minor. Verbal informed consent was sought and granted by participants in the qualitative study to limit mistrust among participants (48). All study participants received an explanation about the purpose of data collection and their rights to drop out, stop the interview, or not answer any questions asked by the researcher as they wished. Collected data were anonymized and stored in password-protected devices to ensure confidentiality for participants.

## Results

### Study Participants

In the qualitative strands, a total of 90 ICs, 86 IDIs and 13 GIs were conducted. In addition, 70 observation records were included. Participants were women and men from different ethnicities (Kinh, Cham, Ra-glai) and age groups such as children, adults, and elderly. Adult participants had diverse profiles including farmers, plantation and forest workers, teachers, forest guards, public health officials, private and informal health providers, local officials, traditional leaders, and respected individuals in the community.

In the quantitative strands, 1,957 HHMs and 410 HHRs participated in the survey (Table 1). Survey participants accounted for 42% (410/973) of the total of households registered in the local census in 2017, mainly representing people who stayed in the “new village” (see further explanation in Territoriality). In each village, the surveys were open until no additional households gave consent to participate. Refusal rate was approximately 5% of the sampling frame because of two main reasons: not willing to give a blood sample and/or not willing to meet with health professionals who were from the dominant ethnicity or unknown to community members.

**Table 1:** Characteristics of survey participants.

	**Frequency (n)**	**Proportion (%)**
Household members (*N* = 1,957)		
Sex		
- Male	717	36.5
- Female	1,240	63.5
Median of age (range); IQR: 22 (1; 89); IQR (7; 33)		
Ethnicity		
- Ra-glai	1,939	99.1
- Chu	3	0.2
- Kinh	14	0.7
- Other (Muong)	1	
Occupation (excluding of children and elderly, multiple responses, *N* = 1,164)		
- Sedentary farming (rice, cassava, sugar cane)	730	62.7
- Slash-and-burn agriculture	1,103	94.8
- Seasonal workers (plantation, construction work)	325	27.9
- Others (grocery owners, trading)	42	3.6
Education (excluding of children aged under 6, *N* = 1,551)		
- Never been to school	590	38.0
- Primary school	632	40.8
- Secondary school	273	17.6
- High school	49	3.2
- University	7	0.5
Household representatives (*N* = 410)		
Sex		
- Male	87	21.2
- Female	323	78.8
Median of age (range), IQR: 37 (16; 71), IQR (27; 44)		
Median of household members (range), IQR: 5 (1; 13), IQR (4; 6)		
Median of forested fields (range); IQR: 2 (1; 8); IQR (1; 2)		

### The Uptake of LLINs and IRS

#### Territoriality

The use of LLINs and the effectiveness of IRS was directly related to population movements and territoriality. Historically, the Ra-glai lived in small clusters of houses in the forest for rotational slash-and-burn agriculture (49). From the 2000s, the government resettled the Ra-glai to so-called *làng mới* or the “new village” located in the lowlands with farmland to cultivate cash crops such as rice, cassava, and sugar cane. Their traditional territory, now referred to as *làng cũ* or the “old village” where their forest fields are, turned into government-controlled forest territory and a hydropower site. Of 410 households, 97.6% owned at least one forest field for slash-and-burn agriculture (Table 2A). To sustain slash-and-burn farming (*n* = 400), 88.0% of HHRs frequently went to their fields, and 48.5% had a plot hut there. Of HHRs who owned the forest field, 38.8% stored their crops there and 25.5% kept animals such as pigs, chickens, and cows at the field. Participants explained that unsuitable soil and limited farming land in the new villages were the reason they returned to the forest field for slash-and-burn farming. Going back to traditional territory additionally meant being closer to the spirits of their ancestors and maintaining the Ra-glai identity and way of life. In the traditional territory, farmers had various economic activities including planting maize, beans, or vegetables, as well as herding cows or goats and collecting forest products (mushroom, orchids, etc.). These combined activities were economically more interesting than planting cash crops around the new villages. Forest farmland ownership and residency were, however, not officially recognized by the local government as they compensated Ra-glai households for the lost houses after the resettlement. Despite these barriers, in one of study villages, up to 25% of households had completely abandoned their new village houses (Figure 1A). A trade-off of this choice was the inability to officially register at the nearest communal administration, limiting access to the government's welfare programs which included favorable loans and healthcare.

**Table 2:** Territoriality, sleeping places, mobility, and malaria status.

	**Frequency (n)**	**Proportion (%)**
2A. Territoriality (household representatives, *N* = 410)		
Households who had at least one forest field	400	97.6
Households who often went to the forest field (*N* = 400)	352	88.0
Households who owned a plot hut at the field (*N* = 400)	194	48.5
Households who kept animals at the field (*N* = 400)	102	25.5
Households who stored their harvested crops at the field (N=400)	155	38.8
2B. Housing, sleeping places, and activities outdoors (household representatives, *N* = 410)		
Households who owned a house in the new village		
- Yes	402	98.0
- No	8	2.0
Housing styles in the new village (*N* = 402)		
- Stilt house	184	45.8
- Brick house	52	12.9
- Both brick and stilt-houses	166	41.3
Housing styles in the field (*N* = 194)
- Stilt house	189	97.4
- Brick house	5	2.6
Sleeping places and activities outdoors (household members, *N* = 1,957)		
Participants who often slept in the new village	1,724	88.1
In the new village, participants who slept inside a stilt house (*N* = 1,724)	240	13.9
In the new village, participants who slept after 21:00 (*N* = 1,724)	1,578	91.5
In the new village, activities from 18:00 to sleeping time (multiple responses, *N* = 1,724)
- Watching TV	1,335	77.4
- Drinking outdoors	112	6.5
- Visiting relatives/friends	370	21.5
At the field, participants who often slept before 21:00 (*N* = 685)	667	97.4
At the field, activities outdoors from 18:00 to sleeping time (multiple responses possible, *N* = 685)
- Watching TV	9	1.3
- Drinking outdoors	26	3.8
- Visiting neighbors	104	15.2
- Visiting friends/relatives in the new village	37	5.4
At the field, participants who often got up before 07:00 (*N* = 685)	670	97.8
At the field, activities outdoors before 07:00 (*N* = 685)		
- Fetching water	241	35.2
- Collecting wood	306	44.7
- Collecting food	243	35.8
- Feeding animals	318	46.4
- Visiting the field	379	55.3
2C. Mobility (household members, *N* = 1,957)		
Frequency of sleeping in the new village and at the plot hut		
- More often in the new village than at the plot hut	1,272	65.0
- More often at the plot hut than in the new village	28	1.4
- Equal frequency, both at the plot hut and in the new village	657	33.6
Participants who went to the forest (*N* = 1,596, excluding children >6 years)	641	40.2
Participants who slept in the forest (*N* = 1,596)	106	6.6
2D. Malaria status (*N* = 1,957)		
Proportion of participants who had fever symptoms	31	1.6
Positive results confirmed by microscopic screening and rapid diagnostic tests	3	0.2

#### Housing Structure and Outdoor Activities

There were two main housing styles: government subsidized brick houses and traditional stilt houses, with the latter made of wood, bamboo, and forest leaves. In the new village, 98.0% of HHRs (*N* = 410) said they had a house there (Table 2A). Of these new village houses, 45.8% reported to have a brick house, 12.9% had a stilt house, and 41.3% had both (Table 2B). Brick houses were identical in style (mimicking the Kinh housing style) and size (24 m^2^), featured brick walls, cement floors, tin roofs, and closable windows (Figure 1A). The Ra-glai could not afford buying beds and often slept on the floor inside the brick house. They sometimes slept in hammocks outdoors for part or most of the night (especially during the dry season) as the brick houses trapped heat inside. Additional stilt houses were frequently built next to brick houses (Figure 1B) to increase sleeping spaces and ventilation. Of HHMs (*N* = 1,724), 13.9% slept in the stilt house while staying in the new village; 13.3% of HHMs who slept in the stilt house said they slept after 21:00. HHMs reported on various number of evening activities, defined as social activites taking place after 18:00, including watching TV (74,8%), drinking outdoors (7,3%), and visiting relatives or friends (21.2%).

Stilt houses were preferred by households as they are made of locally available construction materials, easily cleaned and maintained, and have open structures, allowing to cook indoors (Figures 1C–E). At the forest field, 97.4% (*N* = 194) of plot huts were on stilts (Table 2B). Households could not build brick houses in the forest without having ownership of the land. While staying at the plot hut, they preferred hammock use both indoors and outdoors, to maintain some privacy (a typical plot hut was about 10 to 15 m^2^). At the forest field, 97.4% (*n* = 685) of HHMs slept before 21:00 and 97.8% got up before 07:00. Early morning activities outdoors (defined as all activities before 07:00) were reported by several HHMs including visiting the field (55.3%), feeding animals (46.4%), and collecting water (35.2%), wood (44.7%), or food (35.5%).

#### Mobility Patterns

Complicated mobility between the forest field and the new village, with the purpose of slash-and-burn agriculture in the old village and social and economic activities in the new village, resulted in varied sleeping places. 33.6% (*N* = 1,957) of HHMs said they slept at the forest field as frequent as in the new village. Sixty five percent said they slept more often in the new village while 1.4% slept more often at the field (Table 2C). 40.2% (*N* = 1,596) of HHMs reported to carry out subsistence forest activities and 6.6% stayed overnight in the forest. Young children, from the age of six onwards, were taught to carry out forest activities such as herding cows, guarding the crop against wild monkeys, fetching water, and picking vegetables for daily meals. More than 50% of households listed in the census were not present in the new villages during the two months the surveys took place. People explained that a drought had occurred early in the year destroying the most important crops of that time of year. This drove these households to live in their forest field to assure the second round of crops in the year. The drought also drove many farmers to take on agricultural work in other districts of Ninh Thuan and Lam Dong province (about 100 km away), leaving households short of labor for slash-and-burn agriculture. This necessitated longer stays in the field to get the work done, with less frequent commuting to the new villages.

“*Our priority is to keep our land, our village, and our good traditions. We need to continue our traditional slash-and-burn farming. We want to keep farming in the mountains. People cannot live far from the mountains and the forest. In fact, we would die if we are taken out of the mountains and the forest. We are not familiar to sedentary agriculture; we do not know how to work with water buffalos, but we are experts in mountainous farming. This is what people want in their heart and their mind, but people do not dare to speak out. Because the government already resettled us, they gave us concrete houses and farmland in the lowland. We already received compensation money from the government. But really people do not want this life. We want to go back to the mountains.” (IDI, a Ra-glai traditional leader)*.

#### LLIN Coverage and Use per Location

Sufficient LLIN ownership, defined as having one LLIN per two persons, was reported to be higher in the new village houses (57.0%, *N* = 402) than in the plot huts (5.7%, *N* = 194) (Table 3A). When staying overnight at the forest field, HHMs (*N* = 106) reported bringing different materials for sleeping, including hammocks (69.8%), bednets (66.0%), blankets (3.8%), plastic sheets (39.6%), and extra clothes to wear at night for warmth (7.5%). Additional cotton and polyester hammocks were observed to be used for sleeping at the plot hut, due to a lack of space indoors. When spending the night in the forest for hunting-gathering activities, people said they could not use LLINs because there was no space for hanging them up.

**Table 3:** The uptake of public health services.

	**Frequency (n)**	**Proportion (%)**
3A. LLIN coverage and use		
LLIN coverage in the new village (*N* = 402)		
- No bednet	19	4.7
−2 persons per one bednet	229	57.0
- More than 2 persons per one bednet	162	40.3
LLIN coverage at the plot huts (*N* = 194)		
- No bednet	100	51.5
−2 persons per one bednet	11	5.7
- More than 2 persons per one bednet	83	42.8
Materials brought to the field for sleeping (multiple responses, *N* = 106)		
- Blanket	57	53.8
- Bednet	70	66.0
- Hammock	74	69.8
- Plastic sheet	42	39.6
- Other (extra clothes)	8	7.5
Frequency of sleeping under a LLIN last week (N=1,957)		
- Often	1,708	87.3
- Sometimes	126	6.4
- Not at all	123	6.3
Sleeping under a LLIN the night before the survey (*N* = 1,957)		
- Yes	1,803	92.1
- No	154	6.9
3B. Awareness of malaria (*N* = 410)		
Having heard of “*sốt rét”* or malaria	270	65.9
Selected symptoms that made one suspect of malaria		
- Chills	116	42.9
- Fever	148	54.8
- Sweaty	26	9.6
- Shivering	69	25.6
- Headache/nausea	52	19.3
- Tired, loss of appetite	18	6.7
- Do not know	70	25.9
- Others (sequential hot and cold, feeling hot etc.)	25	9.2
- No answers	2	0.7
Thought that malaria can be cured	237	57.8
Treatment options for malaria (multiple responses, *N* = 237)		
- Doing nothing, malaria can be cured naturally	211	89.0
- Taking non-malarial pills purchased at a grocery shop	34	14.3
- Making a ritual sacrifice performed by a shaman	23	9.7
- Receiving an injection or intravenous drips	49	20.7
- Taking antimalarials at a public health facility	231	97.5
3C. The use of public health services (*N* = 1,957)		
The use of public health facilities for recent illness		
- Yes	1,231	62.9
- No	620	31.7
- Do not remember	14	0.7
- Did not get sick	92	4.7
Reasons to seek care at a public facility (*N* = 1,231)		
- Fever	243	19.7
- Suspected malaria	39	3.2
- Other illnesses	804	65.3
- Do not remember	145	11.8
Median traveling time between home and the health facility (*N* = 1,231): 60 min (min = 1 min, max = 6 h)		
Diagnosed as positive malaria in the recent visit (*N* = 1,231)	76	6.2
Malaria fever improved after taking antimalarials for a number of day (*N* = 76)		
−1 to 2 days	71	93.4
−2 to 3 days	4	5.3
- Do not remember	1	1.3
Stopped taking antimalarials after 2 days when fever was improved (*N* = 76)	6	7.9
The use of public health services by age group (*N* = 1,231)		
- Under 4 years (*N* = 292)	180	61.6
−5-14 years (*N* = 689)	350	50.8
−15-40 years (*N* = 651)	454	69.7
−41 years and above (*N* = 325)	247	76.0

Self-reported use of LLINs was high (*N* = 1,957), both during the week before the survey (87.3%) and the night before the survey (92.1%). Multinominal logistic regression shows that the odds of inconsistent LLIN use, defined as participants who said they “sometimes” or “never” slept under a LLIN, were lower among HHMs who “often” slept in the new village than those who “never” or “sometimes” slept in the new village (AOR≈0.5) (Table 4). HHMs who reported sleeping in a stilt house and going to the forest field had higher odds of reporting “sometimes” for LLIN use (AOR = 2.6 and AOR = 1.9, respectively) than those who did not; but this was not the case for HHMs who “never” and “usually” used a LLIN (AOR = 1.0 and AOR = 1.5, respectively). HHMs who practiced slash-and-burn agriculture had lower odds of reporting “never” using LLINs (AOR = 0.6). The model demonstrated no strong evidence to suggest that HHMs who “sometimes” or “never” used LLINs were different from those who “usually” used LLINs in terms of age, sex, or education, except that HHMs who attended high school or had higher education had lower odds of inconsistent bed net use (AOR = 0.1). Estimates of the random effects suggest that household characteristics had a stronger influence on “never” (σ = 2.0) or “sometimes” (σ = 1.4) LLIN use as opposed to “usually” LLIN use. No similar influence was observed at the level of the commune (σ = 1.0 vs. 1.3, respectively).

**Table 4:** Risk factor analysis for inconsistent LLIN use in the last 7 days before the survey using multinominal logistic regression and the random effects for household and commune (AOR, CCI 95%, *n* = 1,925).

	**LLIN use “sometimes” vs. “usually”**		**LLIN use “never” vs. “usually”**	
	**Raw OR**	**Adjusted OR**	**Raw OR**	**Adjusted OR**
Sleeping in the new village “often” vs. “sometimes” and “never”)	0.4 (0.2–0.8)	0.6 (0.3–1.42)	0.3 (0.2–0.7)	0.4 (0.2–0.8)
Sleeping in a stilt house	2.6 (1.5–4.4)	2.6 (1.4–4.8)	1.1 (0.5–2.2)	1.0 (0.5–2.2)
Going to the forested field	2.5 (1.6–3.9)	1.9 (1.1–3.3)	1.6 (1.0–2.6)	1.5 (0.8–2.7)
Slash-and-burn agricultural practice	2.0 (1.3–3.1)	0.7 (0.4–1.3)	0.9 (0.5–1.5)	0.6 (0.3–1.0)
Age (per 10 years)	1.3 (1.1–1.4)	1.2 (1.0–1.4)	1.0 (0.9–1.2)	0.9 (0.8–1.1)
Education (reference never been to a school)				
- <6 years old	0.2 (0.1–0.4)	0.3 (0.1–0.7)	0.4 (0.2–0.9)	0.3 (0.1–0.8)
- Primary	0.5 (0.3–0.9)	0.7 (0.4–1.2)	0.6 (0.3–1.1)	0.5 (0.3–1.1)
- Secondary	0.8 (0.4–1.5)	1.0 (0.5–1.9)	1.3 (0.6–2.5)	1.2 (0.5–2.5)
- High school or higher	0.7 (0.2–2.1)	0.8 (0.2–2.7)	0.1 (0.0–0.8)	0.1 (0.0–0.9)
Sex (female)	0.8 (0.6–1.3)	0.7 (0.4–1.1)	0.8 (0.5–1.3)	0.8 (0.5–1.3)
Standard deviation between communes		1.3 (0.3–3.4)		1.0 (0.1–3.4)
Standard deviation households within communes		1.4 (1.0–1.9)		2.0 (1.5–2.6)

During the ethnographic field work, people explained they knew about the necessity of sleeping under a LLIN to prevent malaria. However, this was not necessarily practiced due to (1) torn bednets, (2) insufficient space inside the stilt houses, (3) the heat inside the brick houses, (4) the lack of motivation at the plot huts where people perceived little mosquito nuisance, and (5) the preference for sleeping in a hammock both indoors and outdoors. Damaged LLINs were discarded, and pieces of LLIN used for fishing, rope, or garden fence were seen in both the old and new villages.

#### Distribution of IRS and LLINs

Twice a year, the local malaria control program provided IRS to all houses in the new village. Households said that fumigating the house helped keep mosquitoes away and decrease their malaria risk. They specifically mentioned the benefit of IRS in protecting children's health. However, IRS was not provided to the plot huts due to the unrecognized residency status of people in the fields, the geographic difficulty in accessing the Ra-glai traditional territory, and insufficient funding to cover these additional logistic and transportation costs. Responding to previous findings on LLIN coverage (14, 30–32), the local malaria control program provided extra LLINs and LLHNs to “forest goers,” a category used by the NMCP to plan for the distribution of LLINs and LLHNs. CHWs were expected to estimate the number of forest goers in their commune. This was a difficult task as farmers feared legal consequences once their forest activities were revealed. In addition, defining the forest-going Ra-glai was challenging, because: (1) almost everyone went to the forest for farming and seasonal work; (2) the number and configuration of people staying at the field constantly changed according to the agricultural cycle, work requirements, and division of labor in the household; (3) the duration of stay at the field ranged from daily visits to weeks or months. In practice, CHWs would give extra LLINs and LLIHs only to those households more regularly living at the field than the new village. As such, the distribution did not include people who did not report their forest activities to the authorities, *e.g.*, people who moved in Ra-glai communities following the matrilineal kinship line and marriage, or people who exclusively lived at the field and therefore without official residency in the new village.

### The Uptake of Public Health Services

#### IEC Campaigns and Health Literacy

To inform the public, the NMCP organized IEC campaigns focusing on taking LLINs and LLHNs for *đi rùng, ngủ rẫy* or “forest going and sleeping at the forest field.” Forest going was considered a sensitive topic because people link mobility to their (now illegally used) traditional territory, while the government makes an association between slash-and-burn agriculture, ethnic minority living the forest, and *lâm tặc* or “illegal loggers and poachers.” Printed posters and banners with malaria prevention measures in Vietnamese language were hung up at commune health centers (CHCs) while similar messages were delivered to people via monthly village meetings. These messages were not entirely understood or accepted by the Ra-glai because of the sensitivity around territory, the high level of illiteracy, and the lack of Vietnamese language proficiency. Mobility and living in the plot huts limited people's access to health IEC as there were no village health workers or meetings organized in the old villages. Similarly, IEC materials that provided information about malaria in Kinh (Vietnamese language) were not perceived to be accessible.

There was no equivalent word in Ra-glai language for *sốt rét* (in Kinh) or “malaria fever.” The Ra-glai used the term *sa-ki* in their own language to imply bodily discomfort and illness, which includes fevers, fatigue, and headache. *Sốt rét* and *sa-ki* were perceived to be undistinguishable, and knowledge of malaria was considered a medical domain of public health professionals. In the survey, 65.7% (*N* = 410) HHRs had heard of *sốt rét* with the two most commonly recognized symptoms being chills and fevers (Table 3B). 57.8% of HHRs considered *sốt rét* could be cured. Effective treatment options were reported to be “natural cure” (meaning doing nothing or letting the body recover without taking any western medicine) (89.0%), over-the-counter medication (not-antimalarials) (14.3%), animistic rituals to appease the spirits (9.7%), and taking injections of antibiotics or intravenous drips containing fluids, vitamins, or minerals (20.7%). Up to 97.5% (N=237) of HHRs answered that antimalarials from a public health facility could treat *sốt rét*, however, 62.0% of them stated taking antimalarials was the only treatment option. Participants explained that knowledge of malaria is a privilege of medical doctors, therefore when someone had fevers, regardless of it being a malaria fever or not, the patient needed “western” medicines to treat the symptoms and spiritual rituals with the help of a shaman to solve the underlying cause of the illness.

#### Early Case Detection With CHWs

In the malariometric survey, 1.6% (31/1,957) of participants had fever symptoms, 0.2% (3/31) of whom, had a positive diagnostic test result (Table 2D). The three malaria cases were from Ra-glai ethnicity and stayed in the old village at the time of the survey. For early case dection, CHWs said that they relied on patients to proactively seek malaria diagnostic tests at the CHC as they had no resources for this. At the time of the study, one CHC received project funding to implement active case detection. Once a month, CHWs tested forest-goers, as a risk group, using RDTs and light microscopic screening. Due to the difficulty in identifying forest-going Ra-glai persons, considering the whole population's forest mobility, CHWs mainly tested people they considered to be living at the forest fields. Forest-goers were requested to be present in the new village for blood sampling, however, response rates for testing were generally low. Bad roads, rainy weather, unavailable motorbikes, or the lack of budget for the fuel to travel to the new village were among the main reasons many Ra-glai did not get tested. CHWs reported male adults to be the most difficult group to target because of the distance to and remoteness of their work locations.

#### Testing and Treatment at Public Health Facilities

Free diagnostic testings and antimalarials were only provided at public health facilities, including the CHC, the district health center (DHC), and the district or provincial hospital. 62.9% of HHMs sought public health services for their most recent illness, with lower use observed among HHMs who were younger than 15 years old (Table 3C). The median reported traveling time between patient's homes and public health facilities was one hour (min = 1 min, max = 6 h). For a majority of Ra-glai with frequent mobility and intensive labor for slash-and-burn agriculture, long distances and waiting times often led to delays in seeking medical care at the public provider, unless the patient suspected having a malaria fever or a severe fever. To receive free medical care, the patient had to present a valid health insurance card which people without official residency status could not get. This constrained access to public health services for people living in the old villages, where the malaria risk is higher. A previous confrontation with public health professionals, who are mainly from the dominant ethnicity, in addition to the perception that malaria control is part of government forest control, led to some people preferring to seek low-cost medicines from the private sector. The private sector mostly consisted of home-based practices of public health professionals, pharmacies, and grocery shops. These providers offered convenient locations where people could buy medicines to treat fevers and discomfort outside of the official working hours. For many households who lived at the field, the economic loss of one day's work due to commuting to and waiting at the public provider outweighed the cost of buying medicines directly from the private sector.

## Discussion

This study shows how the effectiveness of the Vietnamese malaria elimination strategy is directly related to the way in which the program is embedded in the local social context and highlights the need to critically examine the assumptions of the program. Territorial arrangements and human mobility were key factors for the uptake of vector control measures and public health services among the Ra-glai in Bac Ai, Ninh Thuan. Almost a decade ago, research showed that mobility patterns between the new villages and the forest fields, double residency related to slash-and-burn agriculture, limited knowledge of malaria, and low uptake of LLINs among ethnic minorities were the key to malaria persistence in certain hotspots in the GMS (31, 34, 35, 37). These continue to remain relevant to malaria persistence in Vietnam today.

In Vietnam and the GMS, the distribution of LLINs has been considered as a main tool for vector control (4). Despite the provision of LLINs, several studies in the region have reported how mobility patterns, perceptions of malaria risk and user's preferences have limited LLIN use (9, 34, 37, 50–54). In this study, we found that slash-and-burn agriculture and territorial arrangements (*i.e*. accessing the field, practicing slash-and-burn agriculture, sleeping in a stilt house, less frequently sleeping in the new village) were risk factors associated with inconsistent LLIN use. The effects of household characteristics on reported inconsistent LLIN use suggested that (i) living in small-sized stilted houses, (ii) poor ventilation inside brick houses, and (iii) sleeping outdoors, including for (il)legal forest work, are additional risk factors. We did not find evidence that demographic characteristics such as age, sex and most of the education categories had an influence on consistent bednet use. The study implies that the variability in housing and sleeping places continue to challenge the standardized approach for vector control.

The Vietnamese NMCP has implemented early detection and treatment and health campaigns to boost the uptake of testing and treatment. This study illustrates the interplay of several social factors such as forest mobility and dwelling, insensitive health messages, complex inter-ethnic relation and the doctor-patient hierarchy, and the uptake of malaria testing and treatment. Existing malaria studies in the GMS point to different approaches to increase the uptake of biomedical interventions such as scaling up malaria testing in the private sector, community engagement to promote the uptake of mass drug administration, or active community and engagement with forest workers to promote the uptake of malaria prophylaxis (55–58). Some malaria studies highlight trust building with local populations, effective communication with local people about malaria and the intervention, and community engagement as enabling factors for increasing public acceptance and adherence (59, 60). In Vietnam, there is limited evidence on participation of ethnic minorities in malaria interventions, however, some studies suggest poor ethnic minorities used less free-of-charge health services due to inaccessibility and the lack of culturally adaptation and sensitivity of government programs (61, 62). This evidence suggests that ethnic minority groups being targeted by the state are not always receptive to the public health interventions that are part of this endeavor—including malaria control measures. New tools for malaria elimination such as vaccines (63) and new diagnostic tests and treatment regimens for vivax malaria (64–66) are expected to face similar challenges. Further studies are needed to find out how these tools could be best used by marginalized and vulnerable communities.

This study highlights the need to unpack the assumptions on which the local and national malaria control and elimination interventions are based. These assumptions include the expectation that the relocation of the Ra-glai to the new villages with brick and concrete housing would directly result in an assimilated lowland Kinh lifestyle, including abandoning slash-and-burn agriculture, the full adoption of sedentary farming, and also sleeping under a bednet. The “villagization,” or the regroupment of scattered rural populations into villages by governments (67, 68), has proven to be of challenging in several South-East Asian contexts (40, 69) as these programs tend to ignore the complex relationships between local people, natural spaces, and places. In Vietnam, the resettlement program increased poverty rates among resettled ethnic minorities (70). The lack of farmland in the new villages consequently led to several groups of slash-and-burn farmers moving back to their old villages (41). For the Ra-glai, moving back to their traditional territory constitutes access to the necessary resources for subsistence and to a space for maintaining Ra-glai identity. For malaria interventions in these slash-and-burn agricultural settings, the main bottleneck seems to be the incongruency between the populations sleeping at fields and malaria control programs “seeing” only official villages.

## Conclusion

This study shows the interdependence of socio-political/ecological factors and malaria transmission dynamics and the relevance of understanding social factors to increase the effectiveness of public health interventions. Complex human mobility patterns, territorial arrangements, and inter-ethnic relations continue to influence how and to what extent the Ra-glai use vector control tools and public health services. The study highlights the relevance of locally adapted and contextually-informed interventions and the need for further studies on how to engage vulnerable communities in the local malaria elimination strategy.

## Data Availability Statement

For the qualitative strand, the NVivo database with excerpts of the transcripts relevant to the study is available from the corresponding author on reasonable request. For the quantitative strand, the datasets used and/or analyzed during the current study are available from the corresponding author on reasonable request.

## Ethics Statement

The studies involving human participants were reviewed and approved by Ethical Review Boards of the Institute of Tropical Medicine in Antwerp, National Institute of Malaria, Parasitology and Entomology in Hanoi (NIMPE), and the Vietnamese Ministry of Health. Informed consent to participate in this study was provided by the participants' legal guardian/next of kin.

## Author Contributions

KP, XN, and TN: conceptualization and methodology. TN and TS: software and formal analysis. CG, XN, and KP: validation and supervision. TN: investigation, writing—original draft preparation and visualization. XN and KP: resources. TN, CG, and KP: data curation. CG, KP, RG, and XN: writing—review and editing. XN and DT: projection administration. KP and XN: funding acquisition. All authors have read and agreed to the published version of the manuscript.

## Funding

The study was funded by Belgian Directorate of Development Cooperation (DGD) for the Framework Agreement 4. TN is funded with a PhD scholarship from the DGD; CG is funded through a framework agreement between the Belgian Directorate for Development Cooperation and the Institute of Tropical Medicine.

## Conflict of Interest

The authors declare that the research was conducted in the absence of any commercial or financial relationships that could be construed as a potential conflict of interest.

## Publisher's Note

All claims expressed in this article are solely those of the authors and do not necessarily represent those of their affiliated organizations, or those of the publisher, the editors and the reviewers. Any product that may be evaluated in this article, or claim that may be made by its manufacturer, is not guaranteed or endorsed by the publisher.
